# Towards the colonization of Mars by in-situ resource utilization: Slip cast ceramics from Martian soil simulant

**DOI:** 10.1371/journal.pone.0204025

**Published:** 2018-10-11

**Authors:** David Karl, Franz Kamutzki, Andrea Zocca, Oliver Goerke, Jens Guenster, Aleksander Gurlo

**Affiliations:** 1 Fachgebiet Keramische Werkstoffe / Chair of Advanced Ceramic Materials, Technische Universität Berlin, Berlin, Germany; 2 Bundesanstalt für Materialforschung und –prüfung (BAM), Berlin, Germany; Hogskolan i Halmstad, SWEDEN

## Abstract

Here we demonstrate that by applying exclusively Martian resources a processing route involving suspensions of mineral particles called slurries or slips can be established for manufacturing ceramics on Mars. We developed water-based slurries without the use of additives that had a 51 wt. % solid load resembling commercial porcelain slurries in respect to the particle size distribution and rheological properties. These slurries were used to slip cast discs, rings and vases that were sintered at temperatures between 1000 and 1130 °C using different sintering schedules, the latter were set-up according the results of hot-stage microscopic characterization. The microstructure, porosity and the mechanical properties were characterized by SEM, X-ray computer tomography and Weibull analysis. Our wet processing of minerals yields ceramics with complex shapes that show similar mechanical properties to porcelain and could serve as a technology for future Mars colonization. The best quality parts with completely vitrificated matrix supporting a few idiomorphic crystals are obtained at 1130 °C with 10 h dwell time with volume and linear shrinkage as much as ~62% and ~17% and a characteristic compressive strength of 51 MPa.

## Introduction

A promising concept to explore and subsequently colonize the Moon and Mars is in-situ resource utilization (ISRU), the practice of on-site collection, processing, storing and use of native materials encountered in the course of human or robotic space exploration. Early colonization scenarios propose the direct use of the rock covering, loose granular surface media (including dust, soil and broken rock) composed of various oxide minerals and referred to as Lunar and Martian regoliths. The chemical composition of Lunar and Martian regolith (Table 1) makes conceivable the extraction of metals and ceramics. For the smelting of regolith in blast furnaces and bloomeries to produce base metals, the availability of ceramic tools is an important prerequisite. The ISRU approaches towards ceramics include (i) dry consolidation [1–3], (ii) melting [4,5], (iii) self-propagating high temperature synthesis and geopolymerisation [6,7]. For the first of these approaches, which represents the most realistic initial colonisation scenario, loose regolith powders are pressed into bricks and fused by direct compression or sintering [1–3], such bricks could be used for masonry construction [8]. However, dry consolidation routes are often not suitable for ceramic parts with complex shapes. In respect to Mars, despite research efforts, it remains challenging to assess the feasibility of the ISRU approaches discussed above, especially as many of the proposed routes are not used to a great extent on Earth. It is surprising that to this day wet processing of minerals—the oldest and most universal processing route towards earthenware pottery, established around 30,000 years ago, has not yet been discussed for ISRU. In traditional pottery hydrous aluminum phyllosilicates are mixed with water, then molded into a shape, dried, and fired [9]. Here we present the water-based slip casting technology for fabricating various ceramic parts of different complexity (discs, rings and vases) using solely theoretically available Martian resources, i.e. regolith, gypsum and water. We followed a common approach in simulating extraterrestrial regolith and its properties by using regolith simulants [10]; here we apply the Martian regolith simulant JSC-Mars-1A, which is a natural glassy volcanic ash composed of finely crystallized and glassy particles of Ca-rich plagioclase, Mg-rich olivine, Mg-rich pyroxene, Ti-magnetite and nanoparticulate iron oxides and oxyhydroxides known as npOx which are also responsible for Mars’ reddish appearance [11–16]. We chose this simulant as it is the best-established Mars regolith simulant, allowing for good comparability. The second resource needed is gypsum, which was used for the plaster molds in the slip casting presented in this paper, it can be found in gypsum-rich veins which have been detected at various locations in sedimentary rock on Mars [17,18]. The third resource for wet processing of minerals is water, which is found in the Martian atmosphere, subsurface, regolith and polar caps with the overwhelming majority thought to be in the form of ice [19]. Deposits of water ice that can be > 100 meters thick have recently been reported [20]. In addition, since the discovery of recurring slope lineae in 2013 [21] there is ongoing scientific debate whether there even is contemporary water activity in the form of liquid water brines in shallow Martian soil.

**Table 1:** Chemical composition of the Martian regoliths and the JSC-Mars-1A regolith simulant.

Compound	Regolith			JSC-Mars-1A Martian regolith stimulant	
	Utopia Planitia *	Ares Vallis Mermaid Dune **	Columbia Hills of Gusev crater ***	Orbitec data sheet ****	Authors’ anlaysis *****
SiO_2_	43	50.2	36.1	43.5	37.27
Al_2_O_3_	7	8.4	2.56	23.3	20.74
FeO	n.a.	17.1	15.4	n.a.	n.a.
Fe_2_O_3_	17.8	n.a.	4.84	15.6	14.71
MnO	n.a.	n.a.	0.37	0.3	0.24
MgO	6	7.3	21.6	3.4	3.2
CaO	5.7	6.0	1.69	6.2	5.46
Na_2_O	n.a.	1.3	1.0	2.4	2.07
K_2_O	< 0.15	0.5	0.03	0.6	0.48
TiO_2_	0.56	1.3	0.22	3.8	3.16
P_2_O_5_	n.a.	n.a.	0.39	0.9	0.72
Cr_2_O_3_	n.a.	n.a.	0.63	n.a.	n.a.
SO_3_	8.1	5.2	2.36	n.a.	n.a.
Cl	0.5	0.6	0.53	n.a.	n.a.
CO_2_	n.a.	n.a.	12	n.a.	n.a.
Total	89	98.9	99.8	100	100.77

* Viking 2 landing site, XRF [13].

** Mars Pathfinder, APXS (normalized to a sum of 98%) [14].

*** Spirit Rover, APXS data from [15], recalculated to 12 wt % CO_2_ [16].

**** Developed by Allen et al. [11], (XRF volatile-free, normalized).

***** Simulant analyzed as delivered, XRF (volatile-free).

## Materials and methods

The Martian regolith analog (JSC-Mars-1A) in the size fraction < 1000 μm was supplied by the Orbital Technologies Corporation (ORBITEC, Colorado, USA). The chemical composition of JSC-Mars-1A resembles the Martian regoliths analyzed in the course of the Viking lander, Mars and Spirit Rover missions (Table 1).

### Slurry preparation

Two processing routes to produce slurries were tested. In the first route, milling of the raw JSC-Mars-1A material in a swinging mill was followed by fractionation of material between 25 and 50 μm in a sieving tower. Water-based slurries with 51 w% solid load were prepared from this fraction and roll ball milled for 48 hours with 12 mm ZrO_2_ grinding balls. The material had to be added gradually to the container because the fine particles impeded the dispersion of the material. In another approach, the raw material was simply passed through a coarse 500 μm grid sieve without an intermediate milling step, directly poured into the water and roll ball milled in the same manner as in the first route. A commercial porcelain slurry was obtained from Royal Porcelain Factory in Berlin and used as a reference.

### Slip casting

The molds to slip cast rings with an inside diameter of 30 mm and a height of 18 mm were made using casting plaster with a water plaster ratio of 4 to 5. Rings were cast by placing the molds on steel plates and filling them generously with slurry. To obtain comparable wall thicknesses casting time for JSC-Mars-1A rings was set to 4 minutes and 8 minutes for the porcelain slurry. After casting the remaining slurry and steel plate were removed and the ring mold containing the wet ring was rotated for 90 seconds to generate a homogenous inside surface. Finally, casting overlaps were cut from each side using a knife and the rings were left to dry. To produce vases a plaster mold for porcelain vases from the Royal Porcelain Factory in Berlin was generously filled with JSC-Mars-1A slurry and left to cast for 6 minutes (the increased casting time compared with those for the rings was chosen to accommodate the larger vase geometry) with small amounts of slurry added to keep the liquid level. The mold was emptied and rotated for 120 seconds and the casting overlap cut off. After 15 minutes casts were demolded and small casting failures were retouched using a brush and fresh slurry. After the green body had dried retouched areas and mold burrs were sanded using sandpaper with grit sizes of 2400 and 4000.

### Sintering

For sintering three different firing profiles were applied as follows: (i) heating with 1.7 K/min to 1000 °C, no dwell time, (ii) heating with 1.7 K/min to 1130 °C, no dwell time and (iii) heating with 1.7 K/min to 1130 °C with 10 h dwell time; all followed by furnace cooling. The commercial porcelain slurry was sintered with the optimized schedule, i.e. heating with 2 K/min to 1440°C. The final three sintering schedules were chosen after the side-view hot-stage microscopy study with the approach of having one bisque firing schedule (1000°C) and two schedules in the determined sintering range. All sintering runs were performed in a muffle furnace in standing air atmosphere followed by furnace cooling. The weight loss during sintering was determined using a laboratory scale weighting dried slip cast green bodies and sintered parts.

### Methods

X-ray fluorescence (XRF) analysis of JSC-Mars-1A powder was performed using an autosampler PW 2400 sequential wavelength X-ray spectrometer with Rh-anode (Panalytical, Netherlands). Volatile fraction was measured by heating a powder compact prepared from a mixture of 6 g JSC-Mars-1A powder sample and 1.5 g of Wax C (Hoechst, Germany). A melt tablet was prepared by fused beads method with 0.6 g JSC-Mars-1A material fused into a glass with 3.6 g of FX-X65-2 molten flux (Fluxana, Germany) using the high-frequency furnace Rotomelt at 1200 °C. Particle sizes were obtained with a LS 13 320 (Beckman Coulter, USA) laser diffraction particle size analyzer with exchangeable wet (for water dispersed material) and dry (for powder samples) measurement units. The size distributions were determined for the dry as-received JSC-Mars-1A material, two different JSC-Mars-1A slurries with different preparation routes and a commercial porcelain slurry. Rheological properties of slurries were investigated using a Physica MCR 301 rheometer (Anton Paar, Austria) with parallel-plate geometry (25 mm diameter and 0.5 mm gap size) in rotation mode at 25°C. To determine an appropriate sintering schedule for the produced green bodies, side-view hot-stage microscopy (Hesse Instruments, Germany) was performed on cylinders (3 mm width and 3 mm height) from ground raw material, spring pressure hand pressed with a pressure of 1.5 N/mm^2^. The measurements were conducted with 3 repetitions (that all gave similar results) in air with a heating rate of 10 K/min up to 1350°C with 30 minutes holding time, in a tube kiln. The microscope projects the image of the sample, irradiated from the opposite site, onto a digital image processing system that measures geometry changes during heating as well as changes in width, height and area of the projected images. To evaluate further the sintering behavior of the specimens two parameters were applied, i.e the area shrinkage S_A_ and the isotropic volumetric shrinkage S_V_ defined as S_A_(T) = (A_T0_-A_T_)/A_T0_ and S_V_(T) = (V_T0_-V_T_)/ V_T0_ = (S_A_+1)^3/2^–1, where A_T0_, V_T0_, A_T_ and V_T_ denotes the area (A) and volume (V) at the temperatures T_0_ and T, respectively. A helium gas expansion pycnometer Pycnomatic ATC (Porotec, Germany) was employed to determine the powder particle density for the raw JSC-Mars-1A as delivered. The volume shrinkage *B* and density of green and sintered parts was determined by measuring ring masses with a laboratory balance (RC210P, Sartorius, Germany) and ring volume by X-ray computer tomography CT 40 (Scanco Medical AG, Switzerland). These measurements were performed on one ring sample per sintering temperature before and after sintering for each sintering schedule. The accuracy of the obtained density values was verified by measuring dimensions of cast (and sintered) disks with a caliper and calculating the density using their weight. The linear shrinkage A was calculated from the volume shrinkage B, assuming isotropic shrinkage, according to $A=100(\sqrt[{3}]{B/100+1}-1)$ [22]. The porosity *ϕ* of the parts was calculated using ϕ = (ρ_particle_ − ρ_bulk_)/(ρ_particle_ − ρ_fluid_) with the JSC-Mars-1A raw particle density as particle density *ρ*_*particle*_, the bulk density *ρ*_*bulk*_ and *ρ*_*fluid*_ saturating fluid density—the mass loss of the JSC-Mars-1A powder after firing was considered for the porosity calculation. Microstructural analysis was carried out with scanning electron microscopy (SEM) using a Gemini Leo 1530 (Zeiss, Germany) on fresh fracture surfaces of as-slip casted and sintered samples.

Mechanical properties and Weibull analysis were assessed on 20 identical slip cast samples for each sintering schedule by brittle ring test in a RetroLine mechanical testing machine (Zwick/Roell, Germany) at a deformation rate of 100 μm/min [23]. As the calculated tensile strength values are only to be compared with values for materials obtained from similar ring tests, as critically discussed by Hudson the rings made of commercial porcelain were characterized in comparison [24]. Tensile strength σ_*R*_ is determined according to σ_*R*_ = *KP*/(Πr_2_t), where K is the stress concentration factor being a function of the ratio of internal (r_1_) to external radius (r_2_), P—the applied load, and t the thickness of the annulus [25]. To calculate K we adapt the equation for concentric rings: K = r_2_((r_1_ + r_2_)·(3·(r_1_ + r_2_) − t))/(((r_1_ + r_2_)–t)·t^2^) [26]. Evaluating this formula, we found for one of our sintered rings with a ratio r_1/_ r_2_ = 0.841 K value of 233. This is in good accordance to a value of K = 232 for the same ratio read from a diagram in Durelli and also close to a value of K = 226 extrapolated from a table in Batista [27,28], hence confirming the feasibility of our approach.

## Results and discussion

**Fig 1:**
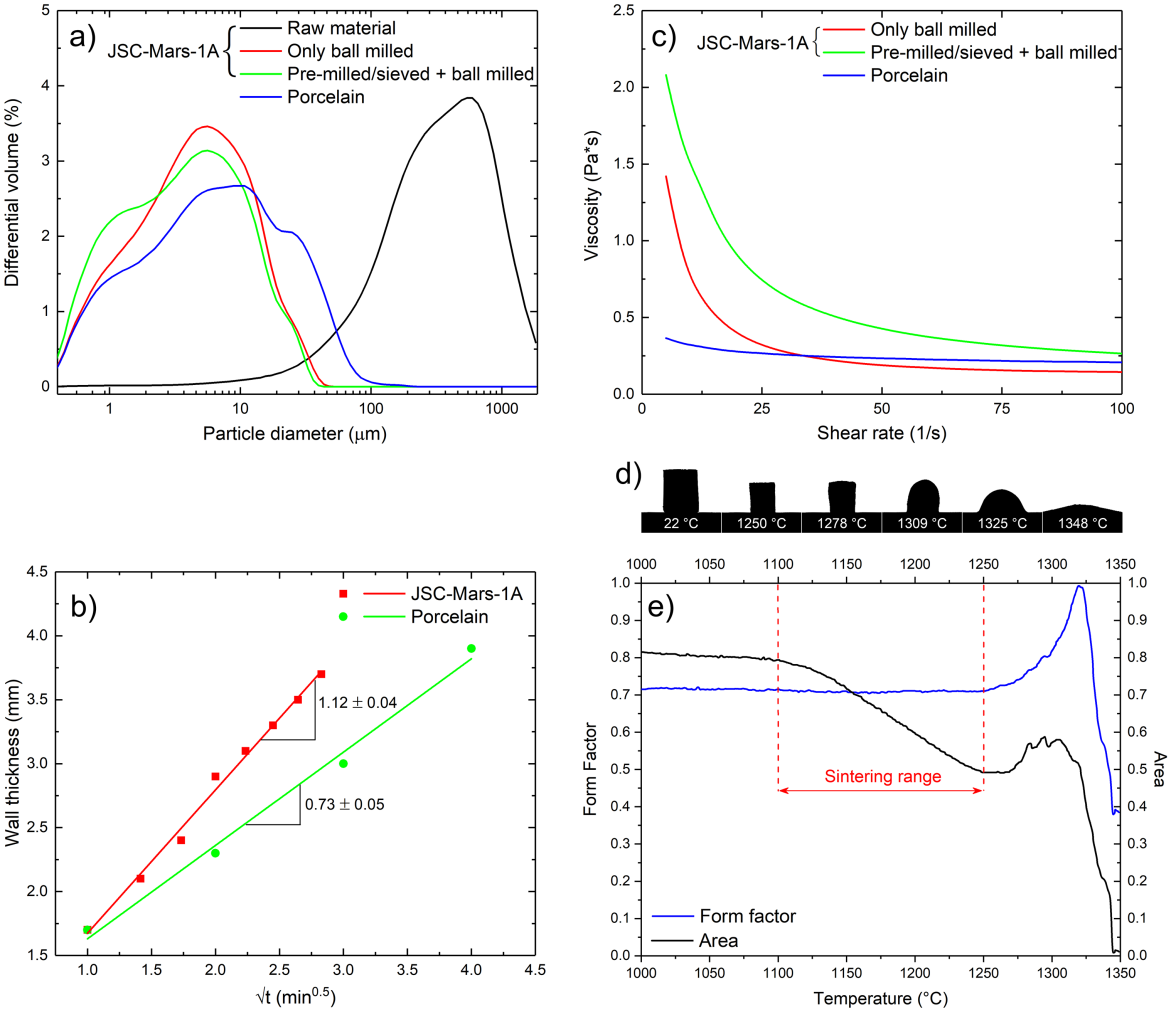
Characteristics of the slurries and sintering behavior of JSC-Mars-1A materials. (a) Particle size distributions in the raw material and in the slurries formulated from differently processed powders in comparison to a commercial porcelain slurry. (b) Viscosities of the slurries in comparison to the commercial porcelain slurry. The small-sized particle fraction in the pre-milled slurries causes a significant rise in the viscosity, making these slurries unsuitable for the casting process. (c) Wall thicknesses of cups slip cast from JSC-Mars-1A and commercial porcelain slurries. (d) Images of JSC-Mars-1A pellets at characteristic temperatures obtained with hot-stage microscopy. (e) The sintering range of JSC-Mars-1A derived from the area of the sample image and the shape of the image.

With future colonization of Mars in mind, our goal was to explore the simplest possible slip casting route to ceramics without any dispersing or binding agents and with a minimum of technological steps. We have found out that neither a milling step nor the addition of additives are necessary for achieving good quality slurries from JSC-Mars-1A regolith simulant. Both particle size distributions and shear viscosity properties underline the suitability of our ISRU-processing route for formulating slurries with processability characteristics similar to those of commercial porcelain slip (Fig 1a and 1b). This finding is particularly relevant, considering that it could potentially allow the processing of slurries with only in-situ resources and avoiding a time and energy consuming milling step. In the next step we produce and characterize three sets of specimens with different complexity and shape, i.e. (i) disks represent the simplest possible geometry, (ii) rings are used for the evaluation of mechanical properties as well as Weibull statistics by brittle ring tests and scaling up our production route by slip casting with a three-part plaster mold to produce a (iii) complex shapes with vase geometry. The drying, demolding and sintering conditions for the JSC-Mars-1A slurries are explored in the next step. By applying the square root of time law we analyzed the overall material transport process during casting (Fig 1c) which is expressed by L^2^∝(Pt/η), where *L* is the layer thickness of the body, *P* the differential pressure across the system, *t* the casting time and *η* the viscosity of the slurry [29]. The slope of the linear fit in Fig 1c is representative for the casting rate, which is indicative of the “rapidness” of the slip casting process. Thicker walls in ceramic components are achieved with the JSC-Mars-1A slurries due to the significantly faster casting (n = 1.12 ± 0.04) compared to an established porcelain slurry (n = 0.73 ± 0.05).

**Fig 2:**
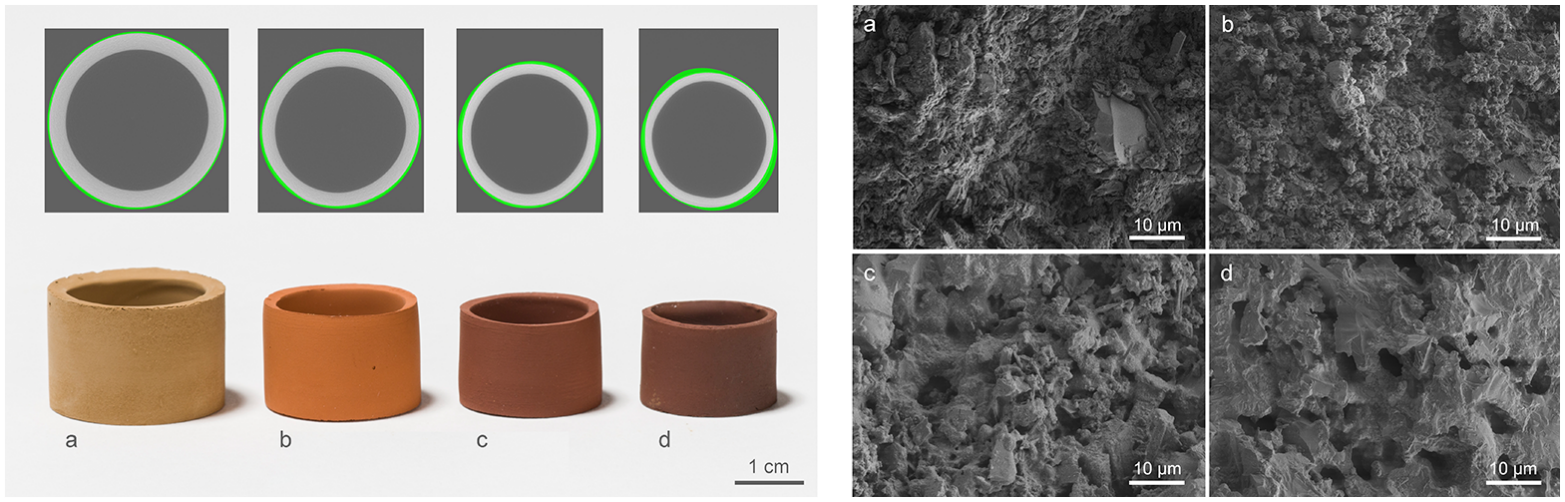
Characteristics of the slip cast parts. Photographic images (left bottom) overlaid with the μCT images (left top) and SEM images (right) of JSC-Mars-1A slip cast rings after demolding (green body) (a) and that sintered at 1000 °C (b), 1130 °C (c) hold 10 h at 1130 °C (d).

The JSC-Mars-1A casts differ from porcelain as they tended to rupture, this holds especially for parts with long planar surfaces. To understand this behavior it is important to note that the regolith simulant does not contain sheet silicates. These phyllosilicates or clay minerals swell upon hydration leading to the special plastic behavior of partially saturated clays. This plasticity is the most important prerequisite for traditional ceramic processing and gives cast porcelain bodies sufficient elasticity to prevent rupture in demolding processes. In contrast, JSC-Mars-1A casts shows no plasticity when still wet and could not be manipulated without breaking. Rupture of the slip cast parts during demolding could be mitigated by increasing wall thickness and using forms with easy to demold surfaces. Hot-stage microscopy is applied to evaluate the sintering regime and set up an appropriate sintering schedule for the slip cast ceramics [30]. Fig 1d and 1e displays the change in the form factor and area of the dry pressed JSC-Mars-1A pellets in the temperature range of interest for sintering, i.e. between 1100°C and 1350°C, underlying several characteristic points. Changes in the form factor correlate with changes in the shape of the sample. The temperature range in HSM experiments in which the area of the samples decreases while the form factor remains unchanged (here 1100–1250°C for JSC-Mars-1A) is the sintering range. The maximum area and volume shrinkage of 0.47 and 0.78, respectively, achievable without deformation of the sample’s shape, is obtained at 1225 °C. The melting begins at 1278 °C as indicated by rounding of the edges of the pellet. Noteworthy (but of minor significance for our present study) are the following characteristic temperatures in HSM measurements: (i) the sphere temperature (1309 °C) at which the edges of the test piece become completely round with the height remaining unchanged. (ii) The hemisphere temperature at which the sample forms approximately a hemisphere (1325 °C), i.e. when the height is equal to half of the base diameter, and (iii). the flow temperature (1348 °C) at which the test piece’ height is one-third of its height at the hemisphere temperature. At temperatures higher than 1250°C, the area of the sample stops decreasing and starts increasing instead. This phenomenon is very well known for porcelain bodies, often referred to as “bloating” [31] and is related to the undesired release and expansion of gases when firing the ceramic at excessively high temperatures. This bloating effect is clearly undesirable and for this reason peak temperatures not higher than 1130 °C were selected for sintering schedules of the JSC-Mars-1A samples. Therefore we set three different firing profiles as follows, sintering at (i) 1000 °C, no dwell time, (ii) 1130 °C, no dwell time and (iii) 1130 °C with 10 h dwell time. All three sintering profiles produced mechanically stable parts with significant differences in shrinkage, mechanical characteristics as well as colors (Fig 2). The best quality parts are obtained at 1130 °C with 10 h dwell time with volume and linear shrinkage as much as ~62% and ~17% (Table 2), respectively, which were significantly greater than the typical shrinkage of phyllosilicate based ceramic material systems such as porcelain.

**Table 2:** Shrinkage (green to sintered), density, porosity and Weibull parameters of slip cast ring samples.

Sample	Shrinkage (volume/linear), % *	Bulk density, g/cm^3^	Mass loss, % *	Porosity, %	Area deviation factor, %	Tensile strength, MPa	Weibull parameter m	Charac-teristic strength, MPa
JSC-Mars-1A, green body	-	1.35	-	62.95	1.75	-	-	-
JSC-Mars-1A, sintered at 1000 °C	30.46 / 9.27	1.44	22.61	62.78	4.39	14 ± 3	4.2	15
JSC-Mars-1A, sintered at 1130 °C	52.36 / 15.07	2.23	22.68	40.35	6.61	36 ± 10	3.8	40
JSC-Mars-1A, sintered at 1130 °C with 10 h dwell time	61.73 / 17.38	2.65	22.76	28.23	8.89	46 ± 11	4.5	51
Porcelain sintered at 1400 °C	-	-	-	-	-	3 ± 6	4.7	41

* from green body to sintered part

**Fig 3:**
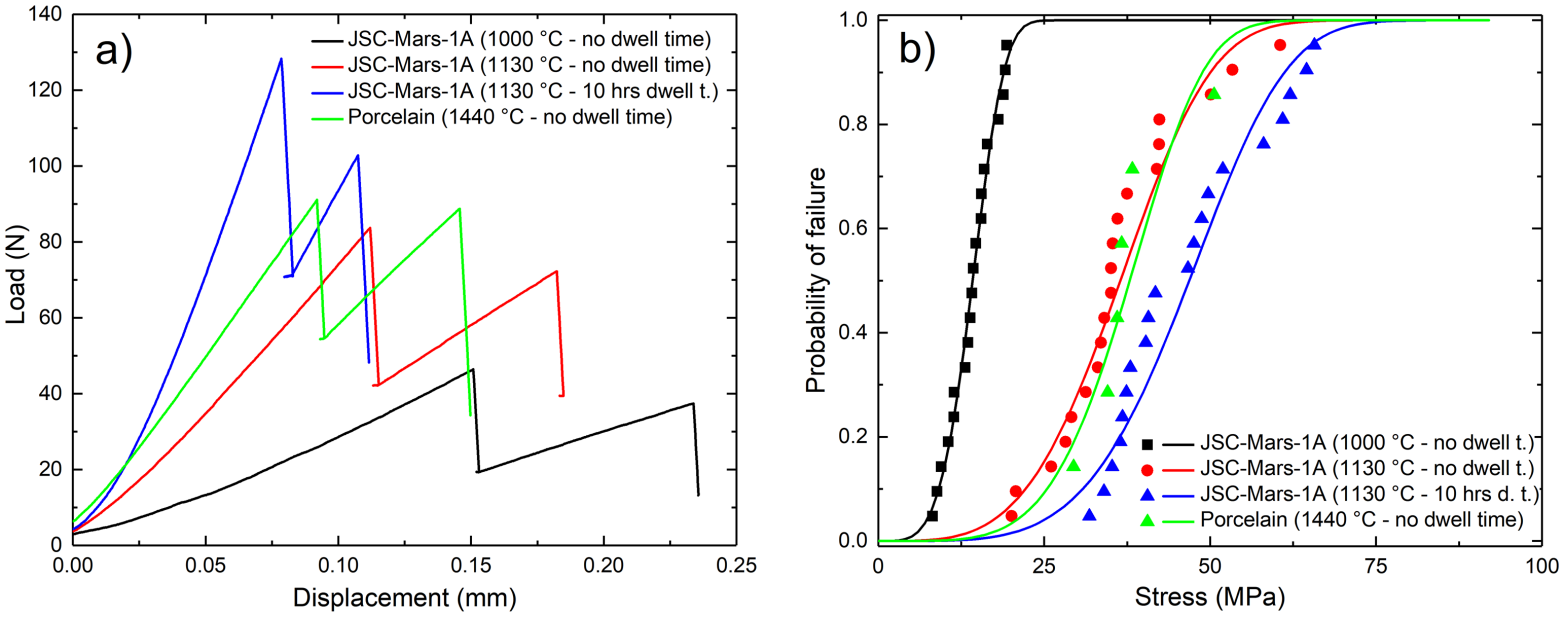
Mechanical properties of slip cast parts. a) Characteristic shape of load-displacement curves obtained by diametral compression (brittle ring test) of four different slip cast ring samples at 100 μm/min. b) Probability of failure as a function of stress (lines), calculated from the Weibull parameters, compared with the experimental values (symbols) and their corresponding probability of failure.

SEM micrographs show that in slip cast green samples larger crystalline grains are embedded in a voluminous but loose matrix of extremely fine particles (Fig 2a). After bisque firing changes in the microstructure seen in SEM (Fig 2b) are not very pronounced. For this temperature treatment the overall porosity (see Table 2) does almost not change (62.95% to 62.78%) while there is an increase in density (1.35 g/cm3 to 1.44 g/cm3) plus a significant volume shrinkage (30,46%) and mass loss (22.61%). With increasing firing temperature (1130°C, Fig 2c) a significant portion of the sample starts to melt, which in turn leads to less pores with increased size. If the peak temperature of 1130°C is maintained for 10h (Fig 2d), the resulting structure is a completely vitrificated matrix supporting a few idiomorphic crystals. In-depth analysis of microstructural and mineralogical evolution during sintering of slip cast parts from JSC-Mars-1A will be the topic of an upcoming publication. Since the sintering treatment seems to have an effect on the form stability, we analyzed the μCT data by dividing each data set from top to bottom into ten sections from which we obtained ten outlines. The outer- and innermost lines from these ten overlapped ring outlines where used to define the area of deviation (green surfaces around the rings in Fig 3). To give an idea of the increasing sintering deformation with higher temperatures, the areas of deviation (green surfaces) where normalized by dividing them though the respective areas of ideal circles that had the averaged inner diameters of all 20 rings at that specific sintering temperature, obtaining a simple area deviation factor (Table 2). While the green body samples showed a deviation of ~1.8%, the value increases with sintering temperature and time—the sample fired at 1130°C for 10 h has a maximum deviation of ~8.9% and the highest degree of eccentricity. As the analysis of the microstructure showed vitrification of the matrix we conclude that this is the result of partial melting / liquid phase sintering of these samples leading to an increased sinter deformation. As the main iron-containing phases are hematite and maghemite, these are likely responsible for the reddish-brown colors. The color change into reddish is associated with the oxidation state of iron in oxides typical for fired earth (oxidation of Fe_3_O_4_ to Fe_2_O_3_) as described by Sherriff et al. for ancient roman pottery [32]. According to X-ray photoelectron and Mössbauer spectroscopic characterization it could not be attributed to the change of the Fe^3+^ fraction in hematite. Fig 3 shows the shape of the load-displacement curves with fracture occurring in a two-step process. During the tests we observed that the first peak is associated with a fracture across the diameter parallel to the loading plane and the second break is due to the transverse diameter of their outer periphery. The relatively low Weibull parameters and high level of standard deviation could be a result of inhomogeneities in the geometry of the rings, on the one hand in the inner surfaces of the rings from the removal of excess slip after the casting process, as well as from sintering deformation. Overall our slip cast ceramics from Martian soil simulant show exceptionally good mechanical properties compared to the porcelain reference. Of the three sintering schedules only the JSC-Mars-1A samples sintered at 1000°C showed a characteristic compressive strength below that of porcelain (15 MPa), while the ones sintered at 1130°C without dwell time where similar to porcelain (40 MPa) and the samples sintered at 1130°C for 10 h surpassed the reference with a value of 51 MPa. This is especially noteworthy as these samples showed a high calculated porosity (28.23%) compared to standard porcelain, which exhibits firing temperature dependent porosity values as low as < 5% [31,33], and the general rule for the bending strength of ceramics being that the strength decreases exponentially with the increase in porosity [31].

**Fig 4:**
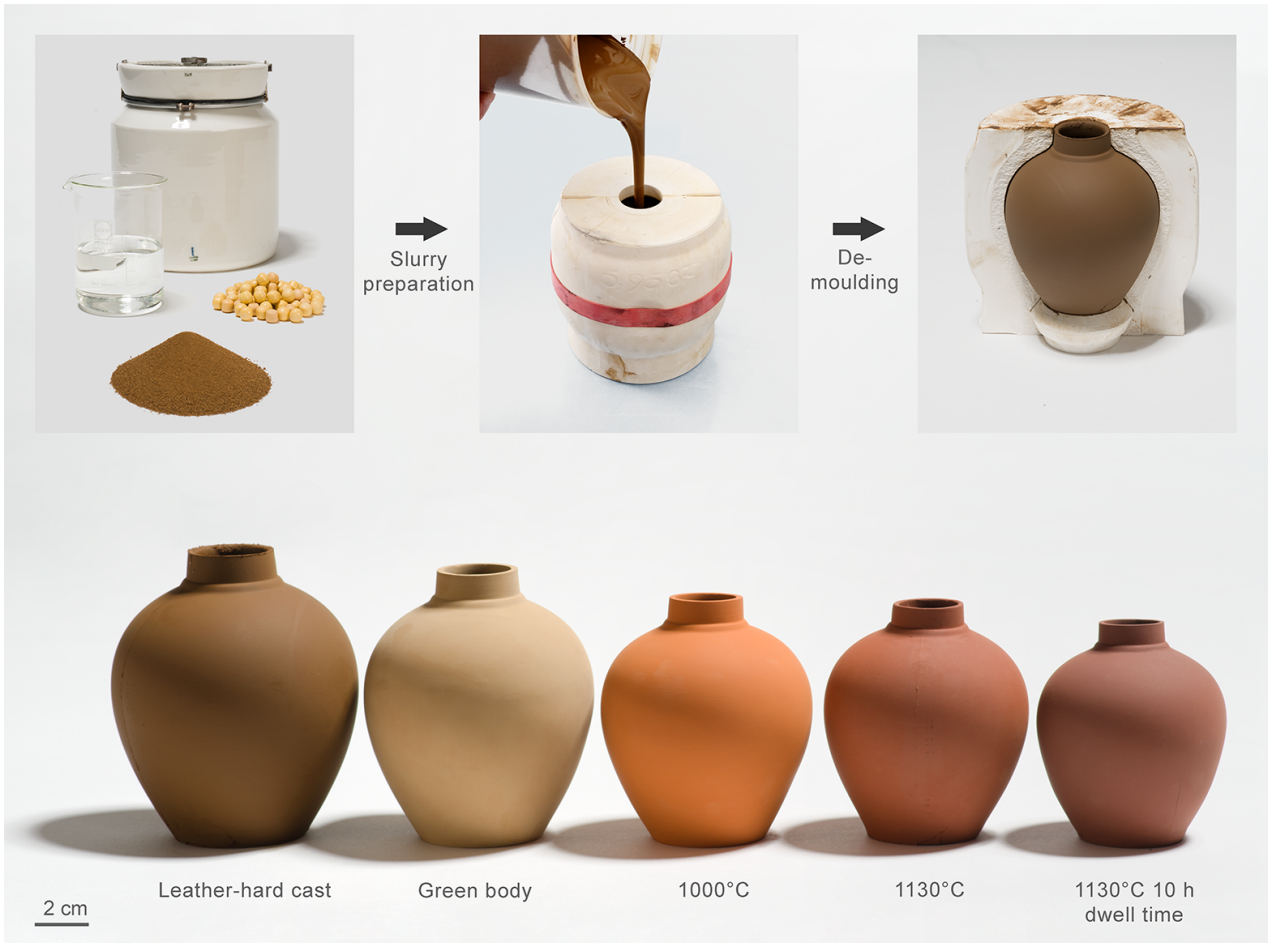
Vases slip cast with the JSC-Mars-1A slurries. Top panel: Slip casting fabrication procedure of vases. Bottom panel: Vases, from left to right: directly after demolding, dried green body, 1000°C without dwell time, 1130°C without dwell time, 1130°C with 10 hours dwell time.

With future colonization of Mars in mind, we verified the applicability of our processing route by using a three-part plaster mold to slip cast a vase geometry (Fig 4). We found the developed casting system to be easily scalable to this bigger size and once we took care not to damage the geometry during the demolding process, we were able to cast intact vases that had a height of 12.5 cm before drying. After being treated in the above developed firing conditions, the vases showed the same mechanically stable characteristics as the simpler ring forms, demonstrating that our simple production route can be scaled to produce stable complex shapes that might be used in possible colonization scenarios of Mars.

## Conclusions

In our work we successfully fabricated mechanically stable ceramic geometries from exclusively Martian resources. Our developed production route is simplistic, yields ceramics that meet the requirements of everyday use and could therefore serve as a starting point for future Mars colonization. We demonstrated that the wet processing of Martian surrogate material via slurries into solid ceramics presents a promising alternative to the multitude of dry consolidation approaches that are presented in literature. Recently there have been reports on the processing of JSC-Mars-1A using additive manufacturing [34,35] and our findings could similarly pave the way for such novel techniques relying on materials in water-dispersed form, such as additive manufacturing technologies like layer wise slurry deposition (LSD) or laser-induced slip casting (LIS) that can be controlled remotely and would enable a production of complex shapes without humans being present on Mars.
